# Influence of a Heterologous (ChAdOx1-nCoV-19/BNT162b2) or Homologous (BNT162b2/BNT162b2) Vaccination Regimen on the Antibody and T Cell Response to a Third Vaccination with BNT162b2

**DOI:** 10.3390/vaccines10050788

**Published:** 2022-05-16

**Authors:** Rieke Reiter, Pia Von Blanckenburg, Reinier Mutters, Julia Thiemer, Reinhard Geßner, Ulf Seifart

**Affiliations:** 1 Institute of Laboratory Medicine, Philipps University Marburg, Baldingerstraße 1, 35043 Marburg, Germany; reinhard.gessner@uk-gm.de; 2 Department of Clinical Psychology and Psychotherapy, Philipps University Marburg, Gutenbergstr. 18, 35041 Marburg, Germany; blanckep@staff.uni-marburg.de; 3 Institute of Medical Microbiology and Hygiene, Philipps University Marburg, Hans-Meerwein-Str. 2, 35043 Marburg, Germany; mutters@staff.uni-marburg.de; 4 Klinik Sonnenblick, Amöneburgerstr. 1-6, 35043 Marburg, Germany; julia.thiemer@drv-hessen.de (J.T.); ulf.seifart@drv-hessen.de (U.S.)

**Keywords:** SARS-CoV-2, booster, third vaccination, BNT162b2, ChAdOx1-nCoV-19, T cell response, antibody concentration

## Abstract

Emerging numbers of SARS-CoV-2 infections are currently combated with a third vaccination. Considering the different vaccination regimens used for the first two vaccine doses, we addressed whether the previous vaccination influences the immune response to the booster. Participants for this prospective study were recruited from among healthcare workers. N = 20 participants were previously vaccinated with two doses of BNT162b2, and n = 53 received a priming dose of ChAdOx1-nCoV-19 followed by a BNT162b2 dose. Participants were vaccinated with a third dose of BNT162b2 in December 2021. Antibody concentrations were determined after vaccination, and in a subset of n = 19 participants, T cell responses were evaluated. Anti-S concentrations and IFNγ production increased during the first 21 days. The choice of the first and second vaccineshad no influence on the final outcome of the booster vaccination. Before booster vaccination, antibody concentrations were lower for older participants but increased more strongly over time.

## 1. Introduction

The spreading of the SARS-CoV-2 Omicron variant has been raising concerns because of its high transmissibility and its potential to infect those previously vaccinated [1,2]. In Germany, due to the declining protection of the BNT162b2 vaccination over time, a third vaccination dose (booster) has been recommended to counter the rapid spread of the Delta and Omicron variants. Booster vaccinations have been associated with reduced COVID-19 mortality [3,4], and have been demonstrated to increase anti-S antibody concentration and neutralizing antibody concentrations, which are highly predictive of protection from symptomatic infection [5], by a factor of 4 to 73 [6]. Moreover, SARS-CoV-2 spike-specific T cell responses are substantially increased in booster recipients [6]. At the beginning of the vaccination campaign, due to a shortage of vaccines and uncertainties regarding side effects, most German citizens received either two doses of BNT162b2 (homologous vaccination) or priming with ChAdOx1-nCoV-19, followed by a second vaccination with BNT162b2 (heterologous vaccination). Studies emphasize the increased effectiveness of a heterologous vaccination regimen compared with a homologous vaccination with regard to the antibody concentration after vaccination [7,8,9,10]. Both vaccination regimens induce CD4^+^ and CD8^+^ T cells reactive to SARS-CoV-2 S-protein peptides [11,12,13]. Studies suggest that these T cells show reactivity against SARS-CoV-2 variants [11,14]. In this study, we aimed to investigate the effect of the previous vaccination regimen (heterologous or homologous vaccination) on a third vaccination with BNT162b2 with respect to antibody concentrations and IFNγ production by T cells. Here, we report the results of the first five weeks of the application observation.

## 2. Materials and Methods

### 2.1. Study Population

The application observation started in December 2021. Participants (n = 75) were recruited from among healthcare employees of the hospital Sonnenblick in Marburg, Germany. All employees of the hospital who received a third vaccination with BNT162b2 (Comirnaty, BioNTech/Pfizer, Mainz, Germany/New York, NY, USA) and were willing to participate were included (Table 1). There were no exclusion criteria.

The age and gender of each participant were recorded as well as information about the first and second vaccination (date and vaccine) and known infection with SARS-CoV-2. Within the framework of quality assurance in the hospital, all employees were previously given the chance to measure the antibody concentration with the same assay as used in the course of this application observation after the second vaccination. The data from the measurement of n = 46 participants were available. In total, the number of study participants amounted to n = 75, aged 21–63 years. N = 15 men and n = 60 women participated in the study. All participants received their first and second vaccination between May and June 2021. N = 20 participants were vaccinated with two doses of BNT162b2 (homologous), while n = 53 participants received a priming dose of ChAdOx1-nCoV-19 (Vaxzevria, AstraZeneca, Cambridge, UK) followed by a second dose of BNT162b2 (heterologous). Two participants received only one dose of BNT162b2 because of previous SARS-CoV-2 infection (positive PCR test result). One participant left the study after day 0, one missed day 21, and another left before day 35. N = 4 participants did not continue after day 7. The study was registered as an application observation by the Paul-Ehrlich-Institute (NIS-No. 642) according to § 4 (23) sentence 3 of the German Medicines Act. Informed consent was obtained from all participants.

### 2.2. Antibody Assay

For antibody measurement, venous blood samples were collected before booster vaccination as well as 3 days and 3 weeks after the booster. For a subset of n = 19 participants, concentrations were also measured 1 and 5 weeks after booster vaccination. Anti-S antibody concentrations were determined using the Elecsys Anti-SARS-CoV-2 S immunoassay (Roche, Basel, Switzerland). The test detects IgG, IgM, and IgA (quantitative), and has a sensitivity of 100% (4 weeks after positive PCR) as well as a specificity of 99.98%. The assay used a cut-off point of 0.8 U/mL to classify samples as reactive (≥0.8 U/mL) or non-reactive (<0.8 U/mL). Anti-S antibody concentrations are reported in U/mL. According to the manufacturer, 1 U/mL is equivalent to 1 BAU/mL (WHO standard). Antibodies against the N-Protein were measured with the Elecsys Anti-SARS-CoV-2 N immunoassay (Roche, Basel, Switzerland), which has a sensitivity of 99.5% (14 days after positive PCR), and a specificity of 99.5%. The test detects IgG, IgM, and IgA (qualitative).

### 2.3. T Cell Assay

The T cell response was measured before as well as 1 and 5 weeks after booster vaccination in a subset of n = 19 participants (Table 2) using the QuantiFERON SARS-CoV-2 assay (Qiagen, Hilden, Germany). Lithium heparin whole blood samples were collected and the assay was conducted following the manufacturer’s instructions. In short, 1 mL of whole blood was added into a nil tube (negative control), a test tube (Ag3, coated with SARS-CoV-2 peptides covering the S, N, and M domains as well as other domains from the full SARS-CoV-2 genome), and a mitogen tube (positive control). Samples were incubated at 37 °C overnight and collected by centrifugation (20 °C, 15 min, 3000× *g*). Plasma was stored at −80 °C until further analysis. To evaluate the SARS-CoV-2-specific T cell response, IFNγ was measured using the QuantiFERON ELISA (Qiagen, Hilden, Germany). The limit of detection is 0.065 IU/mL. A sample was defined as SARS-CoV-2-reactive if a response ≥0.15 IU/mL greater than the background IU/mL value from the QuantiFERON nil tube was measured [15,16,17]. All samples showed a strong response after stimulation using the mitogen tube. 

### 2.4. Statistical Analysis

Participants with either a previous SARS-CoV-2 infection (n = 5) or unknown infection status (anti-N antibodies not measured, n = 6) were excluded from the statistical analysis. Repeated measurements ANOVA with Greenhouse-Geisser correction followed by post hoc testing with Bonferroni correction were conducted to analyze antibody concentrations on day 0, day 3, and day 21. Due to the limited number of participants, data from day 7 and day 35 as well as reported antibody concentrations after the second vaccination were not included in the ANOVA. Instead, significances between the second vaccination and day 0, day 3 and day 7 as well as day 21 and day 35 were calculated using the Wilcoxon matched-pairs signed rank test. Differences between the vaccination regimens were analyzed using the Mann-Whitney U test. In the instance that correlation analysis was conducted, the Pearson correlation was used. The Friedman test and subsequently Dunn’s multiple comparisons test were used to calculate the significances of the T cell response. The analysis was carried out using SPSS 26.0 and GraphPad Prism 9.

## 3. Results

### 3.1. Antibody Concentrations

**Figure 1:**
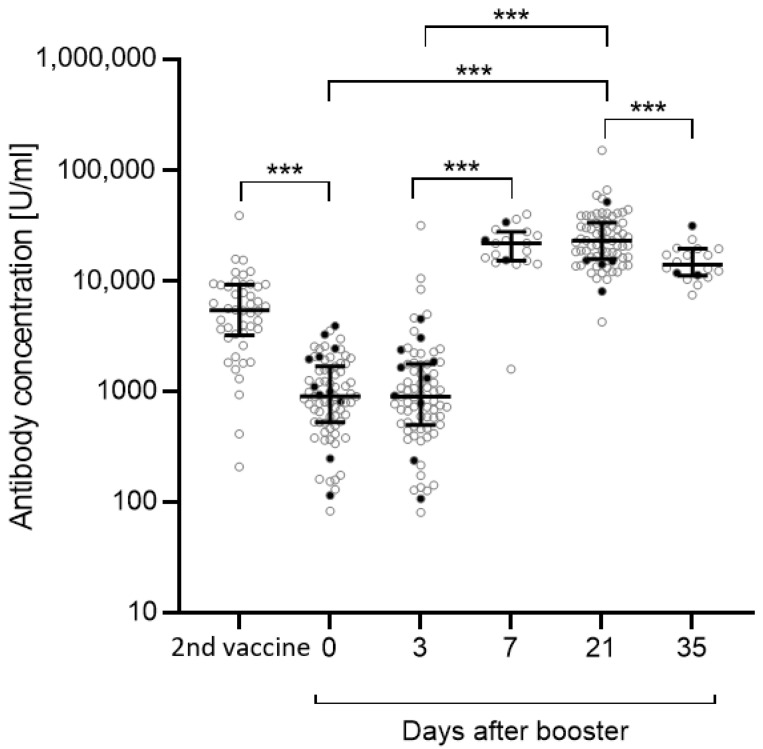
Anti-S antibody concentrations between the second vaccination (May/June 2021) and day 35 post-booster vaccination (January 2022). Anti-S antibody concentrations were measured before administration of the third vaccination, 3 and 21 days post-vaccination, as well as 7 and 35 days post-vaccination in a subset of 19 participants. Antibody concentrations after the second vaccination were available for n = 46 participants. Significance between day 0, day 3, and day 21 was calculated by repeated measurement ANOVA with Greenhouse-Geisser correction followed by post hoc testing with Bonferroni correction. Significances between the second vaccination and day 0, day 3 and day 7 as well as day 21 and day 35 were calculated by Wilcoxon matched-pairs signed rank test. Participants with a previous SARS-CoV-2 infection (n = 5) or an unknown infection status (not tested for anti-N antibodies, n = 6) (black dots) were excluded from the statistical analysis. Data are shown as the median with IQR. *** *p* < 0.001.

The anti-S antibody concentration after the second vaccination in May/June 2021 was known for n = 46 out of n = 75 participants. Between May/June 2021 and the beginning of the application observation, the anti-S antibody concentration significantly dropped (*p* < 0.001) (Figure 1). There was no relevant change in the antibody concentration directly before the administration of the third vaccination and 3 days after receiving that booster vaccination. Between day 3 and day 7 after booster vaccination, the anti-S antibody concentration drastically increased (day 3 vs. day 7: *p* < 0.001), and remained stable until day 21 (day 0 vs. day 21: *p* < 0.001; day 3 vs. day 21: *p* < 0.001). Compared with day 21, the antibody concentration declined by about 30% at day 35 (day 21 vs. day 35: *p* < 0.001). In total, 5 out of n = 75 participants either presented a positive PCR test result (n = 2) or tested positive for anti-N antibodies (n = 3). N = 6 were not tested for anti-N antibodies because they left the study.

### 3.2. T Cell Response to the Third Vaccination

**Figure 2:**
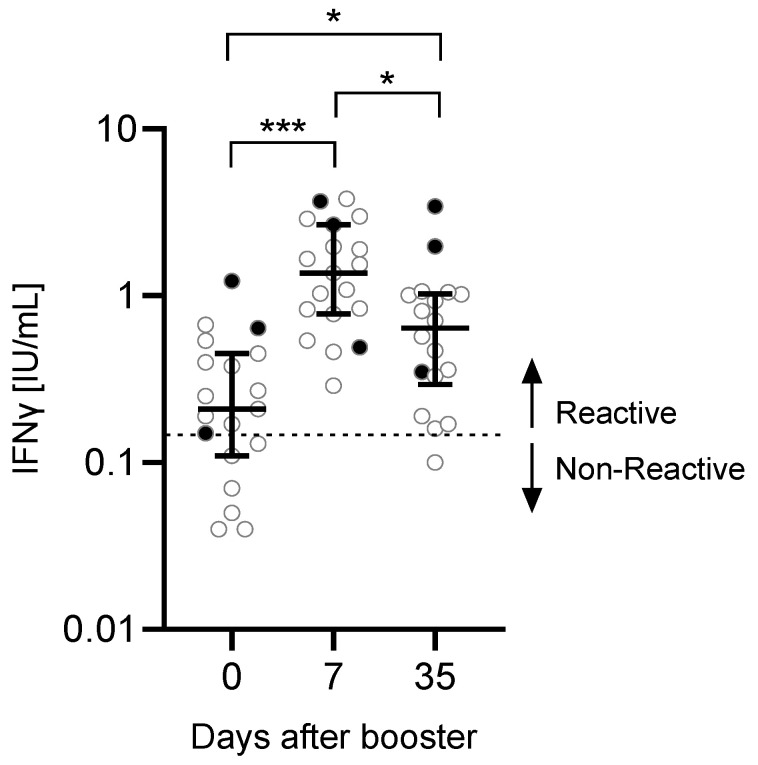
IFNγ production after booster vaccination. In a subset of n = 19 participants, SARS-CoV-2-specific T cell responses were measured by QuantiFERON assay. The read-out parameter was the IFNγ production. Significance was calculated using the Friedman test followed by Dunn’s multiple comparisons test. Participants with a previous SARS-CoV-2 infection (n = 2) or unknown infection status (n = 1) (black dots) were excluded from the statistical analysis. Data are shown as the median with IQR. * *p*: 0.05–0.01, *** *p* < 0.001.

Before administration of the booster vaccine, 6 out of 19 participants were negative for SARS-CoV-2-reactive T cells (measured IFNγ response <0.15 IU/mL) (Figure 2). However, SARS-CoV-2-reactive T cells were found in all tested participants 7 days after the booster. One participant had a non-reactive response 35 days after the booster. 

After the third vaccination, the IFNγ production in response to SARS-CoV-2-specific stimulation increased significantly comparing day 0 with day 7 (*p* < 0.001) or day 35 (*p* = 0.0185). There was a significant reduction in the IFNγ production between day 35 and day 7 (*p* = 0.0411). No correlation was found between anti-S concentrations and T cell response (day 0: r = −0.3662, *p* = 0.1630; day 7: r = 0.07734, *p* = 0.7759; day 35: r = −0.1290, *p* = 0.6469).

### 3.3. Influence of the Vaccination Regimen on the Antibody and T Cell Response and Influence of Gender and Age

**Figure 3:**
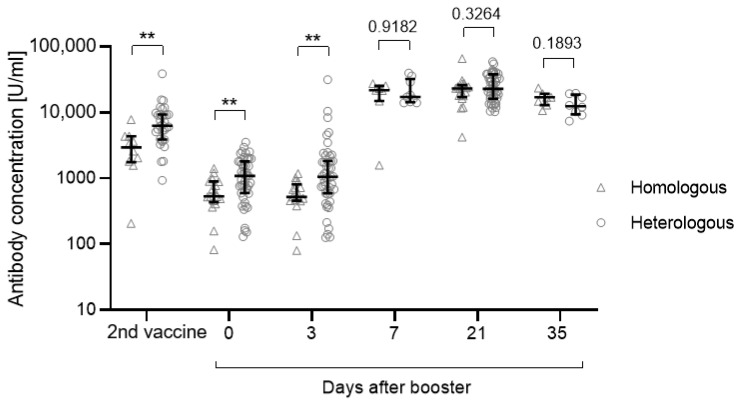
Anti-S antibody concentrations depending on the vaccination regimen (heterologous or homologous vaccination). In May/June 2021, n = 17 participants received two doses BNT162b2 (homologous), while n = 47 participants were primed with ChAdOx1-nCoV-19 followed by a second dose of BNT162b2 (heterologous). Significance was tested using the Mann-Whitney U test. Participants with a previous SARS-CoV-2 infection (n = 5) or an unknown infection status (not tested for anti-N antibodies, n = 6) were excluded from this analysis. Data are shown as the median with IQR. ** *p*: 0.01–0.001.

After the second vaccination, the anti-S concentration was significantly higher for those who underwent a heterologous vaccination regimen compared with those who received a homologous vaccination (*p* = 0.0018) (Figure 3, Table 3). This difference was still significant before administration of the third vaccination and 3 days after (day 0: *p* = 0.008; day 3: *p* = 0.0046). After day 3, antibody concentrations increased independently of the previous vaccination regimen (Figure 3).

**Figure 4:**
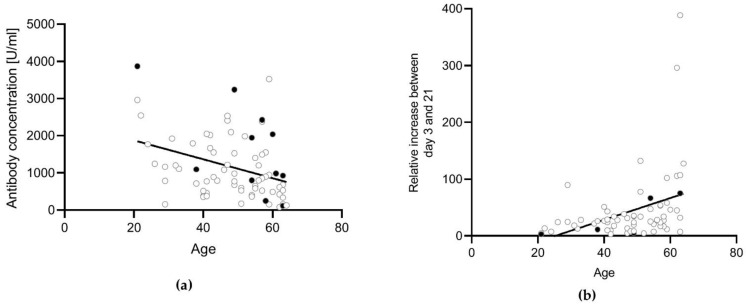
Correlation between age and anti-S antibody concentrations. Correlation analysis was conducted using the Pearson correlation. (**a**) Antibody concentrations at day 0 were correlated with the age of the participants. There was a weak negative correlation detectable (r = −0.3488, *p* = 0.0022). (**b**) The relative percentage increase in the antibody concentration between day 3 and day 21 was correlated with the age of the participants. There was a weak positive correlation detectable (r = 0.3736, *p* = 0.0016). Participants with a previous SARS-CoV-2 infection (n = 5) or an unknown infection status (not tested for anti-N antibodies, n = 6) (black dots) were excluded from the statistical analysis.

Gender did not influence the antibody concentration after the second vaccination (*p* = 0.3832). There was a significantly higher antibody concentration detectable in male participants compared with female participants at day 0 (*p* = 0.0457). However, no difference was detectable on day 3 (0.0916), day 7 (*p* = 0.6806), day 21 (*p* = 0.668), or day 35 (*p* = 0.6126). Age did not significantly influence the antibody concentration after the second vaccination (r = −0.1339, *p* = 0.3752). However, older participants had lower anti-S antibody concentrations directly before the booster vaccination (day 0: r = −0.3316, *p* = 0.0074) (Figure 4a), but a stronger relative increase during the first 21 days after vaccination compared with younger participants (r = 0.3579, *p* = 0.0037) (Figure 4b). Therefore, there was no difference depending on the age at day 21 post-vaccination (r = 0.07655, *p* = 0.5477).

**Figure 5:**
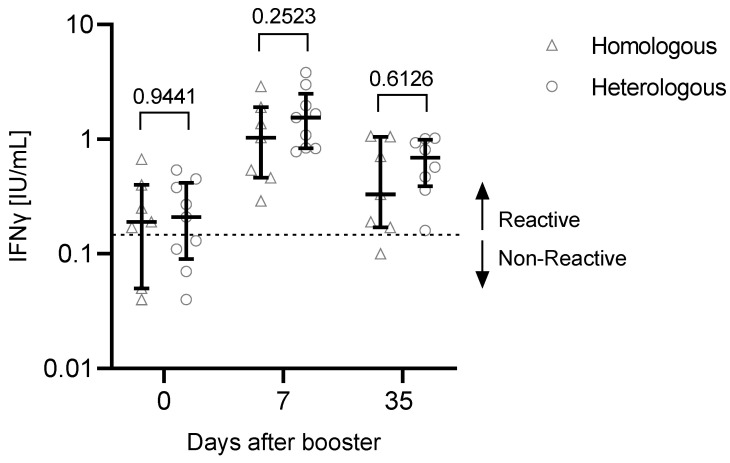
T cell response depending on the vaccination regimen (heterologous or homologous vaccination). In May/June 2021, n = 7 participants received two doses of BNT162b2 (homologous), while n = 9 participants were primed with ChAdOx1-nCoV-19, followed by a second dose of BNT162b2 (heterologous). Significance was tested using the Mann–Whitney U test. Participants with a previous SARS-CoV-2 infection (n = 2) or an unknown infection status (not tested for anti-N antibodies, n = 1) were excluded from this analysis. Data are shown as the median with IQR.

There were no significant observable differences in the T cell response depending on the vaccination regimen (Figure 5, Table 4).

## 4. Discussion

To our knowledge, this is one of the first studies focusing on the antibody and T cell response to the SARS-CoV-2 booster vaccination with BNT162b2 following a homologous or heterologous vaccination regimen. As previously described [7,8,9,10], we observed a significant influence of the vaccination regimen on the anti-S concentration after the second vaccination.

Three important findings were obtained. First, this study showed no influence of the investigated vaccination regimens on the response to a booster dose of BNT162b2. Significant differences depending on the vaccination regimen aligned shortly after administration of the booster (day 7 to day 35). Second, between the second and third vaccination, older participants had a stronger decline in antibody concentration. Third, we did not find a correlation between the T cell response and the antibody concentration on days 0, 7, or 35. Of note, only a small subset of T cell responses was investigated in this study.

In terms of the first finding, there is little information in the literature investigating the effect of a ChAdOx1-nCoV-19/BNT162b2 and a BNT162b2/ BNT162b2 vaccination regimen on the response to a third vaccination. Clemens et al. reported a benefit from choosing a booster vaccine that differs from vaccines used for previous vaccination [18]. We did not observe a stronger booster response for those previously vaccinated with ChAdOx1 nCoV-19 and BNT162b2 compared with those who received two doses of BNT162b2. However, this does not exclude the possibility that another choice of booster vaccine would lead to a stronger response compared with the usage of BNT162b2. BNT162b2 has been described as being especially beneficial as a booster vaccine after the administration of two doses of CoronaVac [19].

Regarding the second finding, a previous study by Nomura et al. reported similar results, with older participants (60 to 70 years) having significantly lower anti-SARS-CoV-2 antibody concentrations 6 months after receiving the second vaccination [20]. Neutralizing antibodies play a special role in the protection against SARS-CoV-2 infection [5]. Even though this study and the literature describe a stronger decline in antibody concentrations of older participants over time [20], other studies have reported that neutralizing antibody concentrations were higher among older patients compared with younger patients [21], potentially compensating for the overall lower antibody concentration in respect to the protection. It remains to be clarified which (neutralizing) antibody concentration is necessary to protect from infection by Omicron or Delta. In contrast to our results, Romero-Olmedo et al. found that responses (antibody levels and T cell responses) were generally lower in older adults after the first and second vaccination with BNT162b2 [12]. Of note, this study focused on a comparison between participants aged 20–53 and participants aged >80 years, whereas in our study no one older than 64 years participated. Interestingly, Romero-Olmedo et al. described how low-/non-responders to the first two vaccination doses could be rescued by a third vaccination resulting in an increase in the antibody concentration.

It is still difficult to define a precise cut-off level for antibody-dependent protection against SARS-CoV-2 infection, especially for virus variants. This is further complicated by the use of many different assays that are currently available and are difficult to compare with each other. To address this problem, the WHO defined Binding Antibody Units (BAU)/mL as an international standard for anti-SARS-CoV-2 spike protein immunoglobulin (NIBSC code 20/136). A study by Feng et al. described that 80% of symptomatic infections can be prevented by an anti-SARS-CoV-2 antibody concentration of 264 BAU/mL [22]. This implies that most participants were still protected from symptomatic disease before the administration of the third dose of the vaccine. However, most studies focus on neutralization assays to describe the protective potential of SARS-CoV-2 spike-protein-specific antibodies [10,11,23]. A previous study has shown that the results of the antibody test used in this application observation align well with the neutralizing activity [24].

A few limitations and possible sources of bias in this study must be considered for the interpretation. First, the participants were limited in number and were all healthcare workers vaccinated at a single hospital. Thus, one should take this into consideration when generalizing the obtained results. Second, most of the participants were female, and participants of an older age were overrepresented. Third, the study reported solely quantitative information. Qualitative factors for the protection against SARS-CoV-2, e.g., cross-reactivity or information about the neutralizing potential of the antibodies, have not been investigated. It has previously been discussed that cross-reactive T cells also react with currently circulating new virus variants [11,14,25]. Finally, the measured IFNγ response does not allow discrimination between contributions of CD8^+^ and CD4^+^ T cell subsets. There are variants of the QuantiFERON assay that was used in this study that allow measurement of the CD4^+^ T cell response and the combined CD8^+^ and CD4^+^ T cell response. A comparison of these two variants results in augmented IFNγ levels when both subsets are stimulated, indicating that both CD4^+^ T cells and CD8^+^ T cells contribute to the detected IFNγ production [15].

## 5. Conclusions

The booster vaccination led to a significant increase of both, the anti-S concentration and the T cell response. However, the level of increase in the humoral and the cellular response was not correlated. Initial differences in the anti-S concentration due to the vaccination regimen fully diminished after the booster vaccination. The same was the case for initial age-dependent differences in anti-S concentrations.

## Figures and Tables

**Table 1:** Participants’ characteristics. Age and gender were recorded. Previous infection: Either positive PCR test result or anti-N antibodies positive. Out of 75 participants, n = 19 were included for the measurement of antibody concentration and T cell response after 7 and 35 days. AB: antibody concentration (U/mL). Heterologous vaccination: ChAdOx1-nCoV-19 and BNT162b2. Homologous vaccination: BNT162b2 and BNT162b2. IQR: Interquartile range.

Variable	
Gender: m/f	15/60
Vaccination: Homologous/Heterologous/ COVID-19 + vaccination	20/53/2
Previous infection: yes/no/unknown	5/64/6
	Median (IQR)
Age	51 (41–58)
AB after second vaccination	5365 (3281–9024)
AB day 0	895 (524–1609)
AB day 3	890 (500–1743)
AB day 21	22,801 (15,990–33,055)

**Table 2:** Characteristics of participants included for measurement of T cell response. Age and gender were recorded. Previous infection: Either positive PCR test result or anti-N antibodies positive; unknown: anti-N antibodies not determined. Out of 75 participants, n = 19 were included for the measurement of antibody concentration and T cell response after 7 and 35 days. AB: antibody concentration (U/mL). Heterologous vaccination: ChAdOx1-nCoV-19 and BNT162b2. Homologous vaccination: BNT162b2 and BNT162b2. IQR: Interquartile range.

Variable	
Gender: m/f	5/14
Vaccination: Homologous/Heterologous/ COVID-19 + vaccination	7/10/2
Previous infection: yes/no/unknown	2/16/1
	Median (IQR)
Age	43 (41–56)
AB after second vaccination	4950 (3705–8524)
AB day 0	1110 (711–1609)
T cells day 0 [IFNγ IU/mL]	0.21 (0.12–0.425)
AB day 3	1054 (669–1779)
AB day 7	21,503 (15,156–26,486)
T cells day 7 [IFNγ IU/mL]	1.37 (0.805–2.32)
AB day 21	18,319 (14,254–26,486)
AB day 35	13,853 (11,278–18,707)
T cells day 35 [IFNγ IU/mL]	0.64 (0.335–1.018)

**Table 3:** Participants’ characteristics depending on the received vaccination regimen. Participants with a previous SARS-CoV-2 infection (n = 5) or unknown infection status (anti-N antibodies not measured, n = 6) were excluded. AB: antibody concentration (U/mL). Heterologous vaccination: ChAdOx1-nCoV-19 and BNT162b2. Homologous vaccination: BNT162b2 and BNT162b2. IQR: Interquartile range.

Variable	Homologous Vaccination	Heterologous Vaccination
	17 participants	47 participants
Age, median (IQR)	55 (49–60)	47 (40–57)
Gender: m/f	2/15	11/36
AB second vacc., median (IQR)	2948 (1875–4169)	6306 (3913–9293)
AB day 0, median (IQR)	532 (458–888)	1087 (623–1780)
AB day 3, median (IQR)	520 (459–723)	1054 (589–1817)
AB day 21, median (IQR)	23,147 (17,884–24,630)	22,957 (16,337–38,068)

**Table 4:** Characteristics of participants included for measurement of T cell response depending on the received vaccination regimen. Participants with a previous SARS-CoV-2 infection (n = 2) or unknown infection status (anti-N antibodies not measured, n = 1) were excluded. Heterologous vaccination: ChAdOx1-nCoV-19 and BNT162b2. Homologous vaccination: BNT162b2 and BNT162b2. IQR: interquartile range.

Variable	Homologous Vaccination	Heterologous Vaccination
	7 participants	9 participants
Age, median (IQR)	55 (49–57)	41 (33–43)
Gender: m/f	1/6	3/6
T cells day 0 [IFNγ IU/mL], median (IQR)	0.19 (0.11–0.325)	0.21 (0.11–0.38)
T cells day 7 [IFNγ IU/mL], median (IQR)	1.03 (0.5–1.635)	1.54 (0.84–1.97)
T cells day 35 [IFNγ IU/mL], median (IQR)	0.33 (0.18–0.88)	0.69 (0.443–0.95)

## Data Availability

The data presented in this study are available on request from the corresponding author.
